# Combined effects of the hedgehog pathway inhibitor LDE225 and nilotinib in a random mutagenesis screen

**DOI:** 10.1186/ar3598

**Published:** 2012-02-09

**Authors:** Tetsuzo Tauchi

**Affiliations:** 1 First Department of Internal Medicine, Tokyo Medical University, Shinjuku-ku, Tokyo 160-0023, Japan

## 

Recent studies have demonstrated that hedgehog pathway is activated in chronic myeloid leukemia (CML) stem cells via up-regulation of Smoothened (Smo), a seven transmembrane domain receptor protein. LDE225 is a small molecule Smo antagonist which has entered Phase I clinical evaluation in patients with solid tumors. We performed a comprehensive drug combination experiment using a broader range of concentrations for LDE225 and nilotinib. Compared with single agents, the combination of LDE225 and nilotinib was more effective at reducing the outgrowth of resistant cell clones. No outgrowth was observed in the presence of 2 μM nilotinib plus 20 μM LDE225. Also co-treatment with LDE225 and nilotinib resulted in significantly more inhibition of growth than treatment with either agent alone in BaF3 cells expressing wt-BCR-ABL and BCR-ABL mutants (M244V, G250E, Q252H, Y253F, E255K, T315A, T315I, F317L, F317V, M351T, H396P). The observed data from the isobologram indicated the synergistic effect of simultaneous exposure to LDE225 and nilotinib even in BaF3 cells expressing T315I. To assess the in vivo efficacy of LDE225 and nilotinib, athymic nude mice were injected s.c. with BaF3 cells expressing random mutagenesis for BCR-ABL mutation. 7 days after injection (average tumor volume, 100 mm^3^), the mice were randomised into four groups (5 mice per group), with each group receiving either vehicle, LDE225 (20 mg/kg; p.o. once every day), nilotinib (30 mg/kg; p.o. once every day), LDE225 (20 mg/kg; p.o. once every day) + nilotinib (30 mg/kg; p.o. once every day). The LDE225 and nilotinib combination more effectively inhibited tumor growth in mice compared to either vehicle- or nilotinib- or LDE225-treated mice. Histopathologic analysis of tumor tissue from LDE225 plus nilotinib-treated mice demonstrated an increased number of apoptotic cells detected by TUNEL staining. To investigate combined effects of LDE225 and nilotinib on primary Ph-positive acute lymphocytic leukemia (ALL) cells, NOD/SCID mice were injected i.v. with bone marrow mononuclear cells from a Ph positive ALL patient. Treatment with LDE225 and nilotinib demonstrated a marked segregation of apoptotic cells in both the central bone-marrow cavity and the endosteal surface. These results suggest that the combination with a Smo inhibitor and ABL TKIs may help to eliminate the Ph positive ALL cells. Taken together, the present study shows that the combination of LDE225 and nilotinib exhibits a desirable therapeutic index that can reduce the in vivo growth of mutant forms of BCR-ABL-expressing cells.

